# New Insights in Bladder Cancer Diagnosis: Urinary miRNAs and Proteins

**DOI:** 10.3390/medsci6040113

**Published:** 2018-12-07

**Authors:** Gökçe Güllü Amuran, Irem Peker Eyuboglu, Ilker Tinay, Mustafa Akkiprik

**Affiliations:** 1 Department of Medical Biology, School of Medicine, Marmara University, Istanbul 34899, Turkey; gokcegullu@gmail.com (G.G.A.); pekerirem@gmail.com (I.P.E.); 2 Department of Urology, School of Medicine, Marmara University, Istanbul 34854, Turkey; itinay@marmara.edu.tr

**Keywords:** bladder cancer, urinary biomarkers, micro RNA (miRNAs), urinary proteins, cholecystokinin receptor pathway, *p53* pathway

## Abstract

Bladder cancer is the 10th-most common cancer worldwide. The diagnosis and follow-up of patients require costly invasive methods and due to these expenses, bladder cancer continues to be one of the expensive malignancies. Early diagnosis is crucial in bladder cancer as it is in other cancers; therefore, non-invasive biomarkers for early diagnosis are very important. In this review, we aimed to focus on the most recent investigations on potential urinary micro RNA (miRNA) and protein biomarkers for bladder cancer diagnosis and their associated pathways. Studies performed by different groups were compiled and the biomarker properties of various proteins and miRNAs in the urine of bladder cancer patients were evaluated. Key studies were obtained by searching keywords “bladder cancer, urinary miRNA, urinary protein, urinary biomarker”. Targets and the pathways of the miRNAs and proteins were analyzed according to mirBase Catalogue and Panther Database. The major pathways that are targeted by aberrantly expressed miRNAs are Cholecystokinin receptor (*CCKR*), *p53*, *Wnt* signaling pathway, and feedback loops. We hereby conclude that urinary micro RNAs and proteins are promising candidates for bladder cancer diagnosis. It should be noted that urine collection, storage conditions, choice of fraction, and normalization strategies should be standardized.

## 1. Introduction

Cancer is the leading cause of death and a major health problem worldwide. According to the Global Cancer Observatory (GLOBOCAN) database, bladder cancer (BC) is the 10th-most frequently detected cancer in both sexes and 9th-most lethal malignancy in men [1]. When detected early in the non-muscle invasive (NMI) stage, the five-year survival rates of BC are >90%; however, the diagnosis of NMI disease requires close follow-up with potential further treatments, given that five year non-muscle-invasive bladder cancer (NMIBC) recurrence rates range from 50% to 70%, and progression to muscle invasive disease range from 10% to 30% [1].

The gold standard for BC follow-up is cystoscopy and it should be performed every three months for the first two years, every six months for the next two years and then every year according to clinical judgment and patient’s preference for muscle invasive bladder cancer (MIBC) patients [2]. Although the sensitivity of cystoscopy is 90% for diagnosis, it is an invasive procedure and small tumors can be missed. The diagnosis and follow-up of patients require costly invasive methods and due to these, bladder cancer continues to be one of the most expensive malignancies [3]. Therefore, the discovery and utility of urine-based non-invasive markers for the diagnosis and follow-up of bladder cancer might help to prevent the patient from periodically experiencing the physical, mental, and economic burden of cystoscopy. Since cystoscopy is considered as the gold standard in the diagnosis, the sensitivity and specificity of candidate biomarkers in the literature are generally evaluated by comparison with cystoscopy. New biomarker detection studies are usually aimed to reach or exceed the sensitivity and specificity of cystoscopy.

Micro RNAs (miRNAs) and proteins that reflect the properties of BC can be found in urine samples of BC patients. There are various studies on urine bladder cancer biomarkers but the relationship between these needs further study [4,5]. There are currently six Food and Drug Administration (FDA) approved tests for clinical use. These are bladder tumor antigen (BTA) stat, BTA TRAK, nuclear matrix protein 22 (NMP22), NMP22 BladderCheck Test, uCyt, and UroVysion tests. The sensitivity of these kits for bladder cancer diagnosis ranges from 57–82% to 74–88% and the sensitivity increases as the stage and degree of cancer increases. These tests are not used for diagnostic purposes alone without cystoscopy, since they cannot beat the cystoscopy with a sensitivity of 98% [6].

Nuclear matrix protein 22 (NMP22) BC test kit is an enzyme-linked immunosorbent assay (ELISA) based kit that detects NMP22 in urine samples of patients, BTA STAT and Trak kits are designed to detect BTA in voided urine samples. The (uCyt+) ImmunoCyt test detects exfoliated BC cells in the urine by using fluorescent monoclonal antibodies against to a high-molecular-weight form of carcinoembryonic antigen (M344) and bladder tumor cell–associated mucins (LDQ10 and 19A11) [7,8]. UroVysion bladder cancer kit detects chromosome 3, 7, 17 aneuploidies and loss of the 9p21 locus via fluorescence in situ hybridization (FISH) in urine specimens [9]. These methods are either highly sensitive or specific, but never both.

miRNAs are small, 20–24 nucleotides long non-protein coding RNA gene products that mediate target messenger RNA (mRNA) degradation or translational repression and they can serve as oncogenes or tumor suppressors. They can be extracted from a wide range of biological samples such as blood plasma, urine, feces, or biopsy specimens and are generally stable and resistant to various storage conditions. Changes in miRNAs can be detected non-invasively and quantified in body fluids using highly sensitive and standardized techniques. Therefore, miRNAs are good candidates for molecular markers of diagnosis, prognosis, and clinical follow-up [10].

In this review, we will focus on urinary extracellular protein and microRNA biomarkers that will open doors to non-invasive procedures for diagnosis and prognosis of bladder cancer. Evidences were achieved by searching PubMed, Medline, Google Scholar, and Web of Science Core Collection using keywords “bladder cancer, urinary miRNA, urinary protein, urinary biomarker”. Experimentally identified targets and pathways were analyzed according to mirBase Catalogue and Panther Database. 

## 2. Urinary Proteins for Bladder Cancer Diagnosis

Several differences were observed among the protein expressions in urine samples of healthy individuals and patients with bladder cancer. Table 1 summarizes the putative BC biomarkers in urine samples. Among them, Apo-A1, IL-8, MMP-9, MMP-10, PAI-1, CD44, VEGF, ANG, CA9, OPN, PTX3, APOE, AGR2, and soluble Fas are considered as candidate biomarkers for bladder cancer [4,5,11,12,13]. Soluble urinary proteins could be measured in the urine supernatant using inexpensive, rapid, and qualitative immunoassays such as ELISA. Most of the studies focus on FDA approved molecules such as NMP22, BTA, UroVysion, ImmuoCyt but there are also some acknowledged biomarkers such as apolipoprotein A4, Anteriorgradient 2, calprotectin, clusterin, coronin-1A, fibronectin, reg-1, stathmin-1, and γ-synuclein [12,14]. 

Anteriorgradient 2 (AGR2) is a cancer related secreted protein mainly found in adenocarcinomas. Anteriorgradient 2 is found ubiquitously in solid tumors and it can be a valuable biomarker in urine or blood for early diagnosis. Noncancerous organs also express, but not secrete AGR2, while cancer cells secrete AGR2. Anteriorgradient 2 is also found on the cell surface of cancer cells. When taken together, AGR2 secretion and cell surface localization are characteristics of cancer, whereas AGR2 expression alone is not. An ideal biomarker should have properties to distinguish cancer cells from healthy cells and AGR2 can do it via localization and secretion. It has been found that normal bladder urothelial cells show uniform immunostaining with moderate intensity for AGR2 [12]. 

Small amounts of AGR2 are secreted by urothelial cells, but it is not detectable in urine, hence the healthy human urine proteome does not include AGR2. Similarly, the blood proteome of healthy individuals does not contain a significant peptide count for AGR2, suggesting little urothelial secretion to the capillaries of lamina propria. Anteriorgradient 2 expression disappears in urothelial carcinoma (UC) and only 25% of primary tumors were observed to preserve AGR2 expression in a cohort of lymph node positive cases. Anteriorgradient 2 secretion and cell surface localization are characteristics of cancer, whereas AGR2 expression alone is not [12].

Urinary protein levels of FGFR3 and Cyclin D3 were analyzed by Western blotting in 321 bladder cancer follow-up patients and 150 healthy control samples. Urine FGFR3/Cyclin D3 expression analysis showed same detection rates with cytology/cystoscopy. The sensitivity and specificity of cytology/cystoscopy was 80% and 84% respectively, and 73% and 90% for FGFR3/Cyclin D3 [13].

Pterin compounds are considered to be biomarkers for various cancers due to their high expression levels in cancer samples. Six pterin metabolites in urine of 35 healthy control samples and 46 bladder cancer samples were analyzed using high performance liquid chromatography (HPLC). Pterin compound concentrations in bladder cancer patients were found to be higher than normal patients, but only two metabolites (xanthopterin and isoxanthopterin) showed statistically significant change between two groups [40]. The same research group found that the urinary APOA1 and APOA2 levels in bladder cancer samples were higher than the levels established in control samples [4,41,42]. Apolipoproteins were analyzed with an immunoassay for multiplexed detection of APOA1, APOA2, APOB, APOC2, APOC3, and APOE, and all six proteins were found to be significantly elevated in urine samples of BC patients when compared to controls (hernia patient volunteers). Immunoassay analysis revealed that all six proteins could be used as an urinary BC biomarker but five of the proteins, not APOE, could be used as an early diagnosis marker [42]. The protein biomarker panel (IL8, MMP-9, MMP-10, ANG, APOE, SDC1, A1AT, PAI1, CA9, and VEGFA) that includes APOE was analyzed in the urine samples of Japanese BC patients. Urinary biomarker concentrations were significantly elevated in BC samples, high-grade and muscle-invasive tumors. APOE, as a single biomarker, was not sufficient for diagnosis of BC, but a 10-biomarker panel enabled to discriminate patients with BC area under the curve (AUC) = 0.892, sensitivity = 0.85 and specificity = 0.81, respectively). A predictive model trained on the larger institutional cohort correctly identified 99% of the cases [43].

Fei and colleagues quantified ORM1 protein in urine samples of 186 bladder cancer patients and found that urinary ORM1-Cr was higher in bladder cancer patients compared to controls (*p* < 0.0001). They suggest that increased level of urinary ORM1 protein might be a useful biomarker for bladder cancer diagnosis [44].

An ELISA based case-control study revealed that the APE1/Ref-1 levels in urine samples could be a valid and reliable biomarker for BC. Urine APE1/Ref-1 levels of 169 Bladder Cancer patients and 108 healthy controls were analyzed and it has been found that APE1/Ref-1 levels were significantly elevated in cancer samples relative to the healthy controls and correlated with tumor grade and stage. Moreover, high APE1/Ref-1 levels were found in samples of patients with recurrence history of BC [45].

Nuclear matrix proteins (NMPs) play important roles in nuclear structure and function, especially during cell mitosis. Nuclear matrix protein 22 has been found to be shed into the urine of patients with bladder cancer at significantly higher concentrations compared to healthy controls [46]. Nuclear matrix protein 22 is a ubiquitous protein, not limited to malignant cells, that can be released into the urine from dead or dying urothelial cells and can subsequently lead to false positive results in response to other benign conditions such as infection, stones, and hematuria. Nevertheless, in the nomograms based on the NMP22 test, the AUC is higher than nomograms based on cytology (0.82 vs. 0.75) [47]. Nuclear matrix protein 22 was found to be useful in deciding between prompt and delayed cystoscopy in a decision curve analysis, but this always depends on the clinician’s threshold for cystoscopy [48].

Bladder tumor antigen is a human complement factor H-related protein that is produced by malignant urothelial cells which interrupts complement activation and is considered to allow tumor cells to escape from immune surveillance [49]. BTA STAT and BTA TRAK (Polymedco, Cortlandt Manor, NY, USA) are FDA-approved tests for the detection of complement factor H and related proteins in voided urine and it is used for bladder cancer follow up in addition to cystoscopy. In pooled analysis of over 2000 patients, overall sensitivities and specificities were reported for *BTA* STAT and BTA TRAK and they were 58%, 71% and 73%, 66%, respectively [50]. Sensitivity of the assays were improved with high stage and grade of tumor, however like in other non-cell based assays, specificity was lower than cytology in most studies, despite the exclusion of benign urologic conditions. Despite being approved by FDA for BC diagnosis, BTA STAT and BTA TRAK cannot stand for either cystoscopy nor cytology [10,11,22,23,24,25].

Recently, 14 urine proteins in voided urine samples and 10-protein bladder cancer biomarker panel, including IL8, MMP-9, MMP-10, SERPINA1, VEGFA, ANG, CA9, APOE, SDC1, and SERPINE1, were analyzed and validated in a large cohort [22,23,24,25]. A multicentre external validation study with a total of 320 subjects also validated that the diagnostic performance of the 10-biomarker panel out performed any single biomarker (AUC = 0.847 (95% confidence interval, 0.796–0.899). After the external validation study, a custom multiplex assay was constructed, and the analytical performance was compared with the data obtained from the individual ELISA assays. The multiplex assay was found to be rapid and had a better performance. A 10-biomarker panel including IL8, MMP-9, A1AT, ANG, VEGFA, CA9, MMP-10, APOE, PAI1, and SDC1 had the greatest diagnostic performance with an AUC of 0.888. Urinary PAI1 was the most accurate single biomarker, followed by IL8 (AUC = 0.8335 and 0.8489 respectively) [16].

The urinary concentrations of 14 biomarkers (IL-8, MMP-9, MMP-10, SDC1, CCL18, PAI-1, CD44, VEGF, ANG, CA9, A1AT, OPN, PTX3, and APOE) were assessed by ELISA. It was the most accurate model for BC diagnosis (sensitivity 92%, specificity 97%), but a combination of three biomarkers, IL-8, VEGF, and APOE, was also highly accurate (sensitivity 90%, specificity 97%). BTA-Trak ELISA test had a sensitivity of 79% and a specificity of 83%, but urine cytology detected only 33% of BC cases [11].

Another multiplex panel analysis of proteins in urine samples of BC, healthy controls and other samples with varying urological disorders revealed that urine concentrations of IL-8, MMP-9 and 10, PAI-1, ANG, and APOE were significantly increased in subjects with BC compared to healthy controls. Urine CA9 was significantly decreased in BC cases. IL-8, MMP-9, PAI-1 and APOE were significantly increased in high compared to low grade tumors as well as in MIBC compared to nonMIBC [18]

The protein concentrations of IL-8, MMP-9, and Syndecan were assessed by ELISA. Urinary protein concentrations of IL-8, MMP-9, and BTA were significantly elevated in BC subjects but multivariate regression analysis revealed that only IL-8 was an independent factor for the detection of BC [25].

Urine and serum matrix metalloproteinase (MMP)-2 and -9, and tissue inhibitor of metalloproteinase (TIMP)-1 and -2 levels were analyzed by ELISA in BC patients. Urinary TIMP-1 levels were found to be significantly higher in high grade BC rather than the low-grade samples (*p* = 0.022), TIMP-1 distribution was found significantly different between the Ta and T1 stage specimens (*p* = 0.040). Serum neutrophil gelatinase-associated lipocalin (NGAL) levels of non-muscle invasive (Ta and T1) tumors were significantly higher than those of muscle invasive (>T1) bladder cancer (MIBC) (32.8 ng/mL vs. 16.2 ng/mL; *p* = 0.029). Receiver operating characteristic ROC curve analysis revealed that urinary TIMP-1 and serum NGAL may be useful non-invasive biomarkers to provide clinical information for bladder cancer disease [51].

Soluble Fas (sFas) protein expression levels in urine samples from pre-operated NMIBC patients were analyzed by ELISA and it was reported that a urinary sFas test might help to identify NMIBC patients that are at risk of tumor recurrence. The authors found that urinary sFas expression level of patients with recurrent disease was significantly higher than those without recurrent disease [38]. Urinary sFas levels and tissue VEGF protein expressions were analyzed in a prior study. They found that urinary sFas levels were significantly higher in patients with UC than in those without cancer and a positive correlation was found between the expression of VEGF protein and the pathological stage/grade in UC tissues (each *p* < 0.05). A significant correlation was also found between sFas levels and VEGF expressions (*R* = 0.882, *p* < 0.05). The authors conclude that sFas and VEGF may together play important roles in the occurrence, progression and diagnosis of UC [37]. Urine sFas was an independent predictor of bladder cancer recurrence and invasiveness in patients who had a history of non-muscle invasive bladder transitional cell carcinoma (TCC), and it outperformed NMP22 [52].

The expression of *HER2/neu* analyzed in serum and urine samples of bladder cancer patients and healthy donors. Mean serum HER2/neu values were similar between the two groups, but urinary HER2/neu levels were significantly higher in BC group. The ratio of urinary HER2/neu to urinary creatinine was found to be significantly higher in BC group. Even though serum and urinary HER2/neu levels were not associated with clinical pathological factors, the ratio of urinary HER2/neu to urinary creatinine levels were significantly higher in high-grade tumors. Likewise, the ratio of urinary HER2/neu and urinary creatinine was found to be significantly higher in patients with bladder cancer and the authors conclude that a larger cohort is needed to be examined for the use of HER2/neu as a tumor marker [53].

N-Myc downstream-regulated gene 2 (*NDRG2*) is associated with cell differentiation and proliferation in various cancers. *NDRG2* expressions were analyzed in urine samples of 127 patients with bladder cancer and 97 healthy controls and in bladder cancer cell lines by quantitative reverse transcription-polymerase chain reaction (qRT-PCR) and Western blotting. *NDRG2* expression was significantly decreased in urine samples of patients with bladder cancer, both at mRNA and protein levels, also lower expressions were correlated with tumor grade and stage. It has been found that *NDRG2* could be a good diagnostic marker for bladder cancer (AUC = 0.888) [54]. 

Collagens were also analyzed for diagnostic and prognostic capabilities. Protein levels of COL4A1, COL13A1, CYFRA21-1, and NMP-22 in urine supernatants were studied and urine levels of COL4A1, COL13A1, the combined values of COL4A1 and COL13A1 (COL4A1 + COL13A1), and CYFRA21-1 were found significantly elevated in patients compared to the controls. Urinary COL4A1 + COL13A1 up-regulation was found to be an independent risk factor for intravesical recurrence [55].

## 3. Urinary micro RNAs for Bladder Cancer Diagnosis

**Figure 1:**
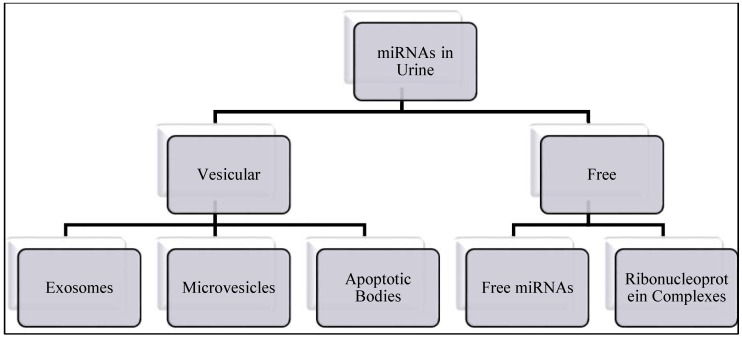
miRNAs in urine could be either as vesicular or free circulating miRNAs.

Micro RNAs are small, around 22 nucleotides long non-protein coding RNAs that regulate gene expression [56,57]. They are synthesized and processed in the nucleus and released into cytoplasm, they can also be found in body fluids as free circulating miRNAs, bound to ribonucleoprotein complexes or in extracellular vesicles (EV), such as exosomes [58]. Figure 1 summarizes the origins of miRNAs in urine.

Since miRNAs can be found in urine EVs and they are protected from degradation, the evaluation of EV miRNA profile represents a promising diagnostic and prognostic non-invasive tool in bladder cancer.

Andreu and colleagues analyzed 43 urine samples (34 bladder cancer, nine non-smoker healthy volunteers). Twenty six miRNAs were deregulated; most of them were significantly down regulated in high-grade bladder cancer patients compared to the healthy group, while only three miRNAs were significantly up regulated in cancer samples. let-7c, miR-30c-2, miR-146a-5p, miR-194-5p, and miR-375 were selected for further validation by qRT-PCR, miR-375 expression was significantly lower in high-grade bladder cancer patients and miR-146a was found to be significantly up regulated in low-grade bladder cancer patients. Non coding small RNAs, Let-7c and miR-194 were slightly down regulated in bladder cancer samples but the difference was not significant. Extracellular vesicle miRNAs were analyzed for recurrence, analysis show that miR-146a, miR-194, and let-7c levels are lower in recurrent low-grade cancer patients, while miR-30c-2 is up regulated in low grade relapsing cases [59]. Although promising, these results are not statistically significant; re-evaluation in a larger cohort is essential. Similarly, the significance of miR-375 and miR-146a expression differences should be verified in a larger cohort. The authors concluded that, if confirmed in a larger cohort, miR-146a could be a useful biomarker for recurrence and differentiation between high and low-grade bladder cancer patients.

Because changes in miRNA expression in cancer tissues exhibit tissue specificity with high stability and detectability, miRNA expression analysis is considered a potential biological marker for both detection and surveillance [57,60,61]. Yun et al. reported that miRNA-200a might have a potential to predict recurrence, however this was not validated in an independent cohort [62]. Sapre et al. reported a six miRNA panel (miRNA-16, -21, -34a, -200c, -205, -221) with an impressive ability to detect active bladder cancer (AUC = 0.85 for the discovery cohort and 0.74 for the validation cohort). The sensitivity of the recurrence estimate was 88%, the specificity was 48% and the panel showed the best performance in T1 tumors [63]. Sasaki and colleagues compared miRNA expressions of bladder cancer cell lines with normal uroepithelial tissue and identified three miRNAs (miR-301b, -563, and -146a-5p) that showed >2-fold higher expression in cancer cell lines. Urine miRNA analysis of these three miRNAs revealed that miR-146a-5p level was significantly higher in urine samples of bladder cancer patients than healthy controls (*p* = 0.0014). Furthermore, the expression of the urine miR-146a-5p in patients with bladder cancer was reduced to the normal level after transurethral resection (*p* = 0.0214). Their results suggested that urinary miRNA-146a-5p levels might be a candidate for non-invasive diagnostic marker for bladder cancer [64]. A decrease in urinary miR-146a-5p after transurethral resection suggests that urinary miR-146a-5p is mostly originated from cancer cells and therefore miR-146a-5p is the diagnostic and prognostic biomarker for urinary bladder cancer.

Recent research revealed that miR-125b, miR-30b, miR-204, miR-99a, and miR-532-3p are significantly down regulated in patients’ urine. A 381 miRNA containing array card was used to analyze urine supernatants of 46 bladder cancer patients and 13 healthy controls. Results were normalized to miR-191, miR-28-3p, and miR-200b, which were selected by the qBase+, the analysis of miR-125 levels provided the highest AUC (0.801) with 95.65% specificity and 59.26% sensitivity, and miR-99a lead to AUC (0.738) with 82.61% specificity and 74.07% sensitivity [65].

It was demonstrated that loss of miRNA-141 and miRNA-200b expression increases the invasion and migration capacities of bladder cancer cell lines and up regulates *MMP-2*, *MMP-9*, vimentin, N-cadherin while down regulates E-cadherin expression. Urine analysis of these miRNAs revealed that miRNA-141 and miRNA-200b expression in urine specimens could distinguish patients with lymph node metastasis from those who were lymph node negative (AUC = 0.704 and 0.674, respectively) [66].

Cell-free miR-214, a secretory miRNA of bladder cancer cell lines, was reduced in urine samples of preoperative bladder cancer patients and increased expression was found in post-operative urine samples. Decreased expression of extracellular miR-214 in urine is associated with a high tumor stage, a high lymph node status, a higher grade, age, and history of NMIBC. The urinary cell free miR-214 can distinguish bladder cancer patients (AUC = 0.838; 95% CI  = 0.796–0.875) from healthy controls and can also serve as an independent prognostic predictor of recurrence-free survival (RFS) and overall survival (OS) in patients with MIBC [67].

Recently, Zhang and colleagues developed a direct PCR method (qRT-PCR-D) for quantification of cell-free miR-155 in urine without RNA extraction. They also tested results with qRT-PCR and found a linear correlation between the two methods. Both methods showed that cell-free miR-155 was significantly increased in NMIBC patients. Cell-free miR-155 RT-qPCR-D distinguished NMIBC patients from cystitis patients and healthy donors with 80.2% sensitivity and 84.6% specificity; this was superior to urine cytology. Cell-free miR-155 was found to be significantly decreased after transurethral bladder resection and its expression correlated with NMIBC stage and grade [68].

To compare the diagnostic performance of a 12 miRNA urine panel with cystoscopy, 81 patients’ profiles were analyzed. A group of six miRNAs including miR16, miR200c, miR205, miR21, miR221, and miR34a, gave the best performance to predict the presence of a tumor (AUC = 0.85). Validation with further 50 patients revealed that miR 16, 21 and 200c, detected in all samples in both cohorts and 6 miRNAs, were detected in a high proportion of samples. miR-16, 200c, 205, 21,221, and 34a were over expressed in bladder cancer samples, expression data normalized to urine osmolality [63].

Eighty five urine samples were analyzed with TaqMan low-density array, 46 miRNAs were selected for validation study in a larger cohort consisting of 121 people, 61 with confirmed BC. Mamm U6 was used as an endogenous control for data normalization [69]. Multivariable analysis shows that a 25-miRNA model could predict the presence of BC with 87% sensitivity and 100% specificity. A 10-miRNA model achieved sensitivity of 84% and specificity of 87% (AUC = 0.902). Limiting the prediction models to 15 and 10 miRNAs resulted in some loss of performance. Several miRNAs (miR-140-5p, miR-199a-3p, miR-93, miR-652, miR-1305, miR-224, miR-96, miR-766) consistently contributed to all models, even though the authors conclude that the most of these core miRNAs have not been reported to be associated with BC previously, this is not the first time that these miRNAs have been associated with cancer [58].

Recently miR-31-5p, miR-93-5p and miR-191-5p were analyzed in urine samples of BC patients and controls and BC patients showed significantly increased expression in urine samples. miR-93-5p, miR-31-5p found to be sensitive enough to detect BC (AUC = 0.81) and expressed significantly different in the various histopathological subgroups of BCA, also decreased post-operatively [70].

Vascular endothelial growth factor-c mRNA and protein expression is increased in tumor tissues, blood and urine samples of BC patients. Conversely, miR-186 expressions were reduced in tumor tissues, blood and urine samples of BC patients. Dual luciferase reporter assay demonstrated that miR-186 binds to 3′-untranslated region of VEGF-C and regulates its expression, indicating that VEGF-C expression is over expressed in BC samples because of miR-186 down-regulation, resulting enhanced invasion and metastasis potential of BC. Direct regulation of VEGF-C by miR-186 indicates that miR-186 could be a good biomarker for BC prognosis in tissue, blood and also urine samples [71].

A comprehensive study by Du and colleagues analyzed and validated the diagnostic and prognostic performance of urinary cell free miRNAs for BC. Urinary miRNAs of BC samples and healthy controls first sequenced with MiSeq, candidate reference miRNAs and differentially expressed ones identified and validated with qRT-PCR. let-7b- 5p and miR-532-5p are defined as the most stable pair of reference miRNAs and 23 other miRNAs defined as differentially expressed ones. Among them, five miRNAs (miR-7-5p, miR-22-3p, miR- 29a-3p, miR-126-5p, and miR-375) were significantly up regulated and two (miR-200a-3p, miR-423-5p) were down regulated in BC samples compared with controls. Results validated in two independent cohorts as training set and validation set. miR-22-3p and miR-200a-3p were identified as independent factors for tumor recurrence in NMIBC. After resection of tumors, miR-22-3p and miR-29a-3p levels in the urine samples were significantly down-regulated, indicating a strong association between miR-22-3p, miR-29a-3p levels and the tumor status [72].

Eight miRNAs; miR-25-3p, miR-18a-3p, miR-92a-3p, miR-140-5p, miR-125b-5p, miR-142-3p, miR-204-5p, and miR-187-3p were analyzed in urine pellets of BC patients, evaluating urinary cell miRNAs as prognostic biomarkers of BC. With a mathematical model of linear combination two miRNA signature, miR-140-5p and miR-92a-3p, identified a subgroup of patients with a higher probability of tumor progression and shorter cancer specific survival [73].

We identified aberrantly expressed microRNAs in bladder cancer urine specimens by literature review [10,58,74,75,76]. We found 106 urinary miRNAs that are associated with bladder cancer (Table 2). Next, we analyzed targets of those miRNAs. According to mirBase Catalogue by Experimental Evidencesthere are 1443 target genes with strong evidence (Supplement 1 file) [77]. Catalogue includes only gene-miRNA interactions that are supported by Reporter assays. 32 of these genes are targeted by more than five miRNAs. Six miRNAs (hsa-miR-100-5p, hsa-miR-122-5p, hsa-miR-155-5p, hsa-miR-29a-3p, hsa-miR-429, and hsa-miR-21-5p) target the same 17 genes, among 32 target genes. 17 common targeted genes and miRNAs are listed on Table 3.

Next, the cellular pathways associated with these 17 genes were investigated by the Panther Database [78]. 17 genes: *BCL2*, *CCND1*, *CCND2*, *CDK6*, *CDKN1A*, *ERBB2*, *IGF1R*, *MYB*, *MYC*, *PTEN*, *RECK*, *RHOA*, *SMAD4*, *SP1*, *TP53*, *VEGFA*, and *WEE1* are components of different cellular pathways. The main pathways and components are CCKR pathway (*BCL2*, *CCND1*, *MYC*, *PTEN*, *RHOA*, *SP1*), p53 pathway, feedback loops (*CDKN1A*, *MYC*, *PTEN*, *TP53*) and Wnt signaling pathway (*CCND1*, *MYC*, *SMAD4*, *TP53*).

Other pathways include angiogenesis, cell cycle, fibroblast growth factor (FGF) signaling pathway, IGF-Mapk cascade, the full list is given in the Supplement 1 file.

Cholecystokinin receptor (CCKR) signaling pathway is triggered by CCK1R and CCK2R, which then influence the expression of downstream genes that affect cell survival, angiogenesis and invasion. p53 pathway regulates the cell cycle, inhibits angiogenesis, and promotes growth arrest, apoptosis, and DNA repair. The Wnt signaling pathway is a signal transduction pathway that pass signals into a cell through cell surface receptors and activates multiple signaling pathways in a cell. Wnt signaling results in transcriptional activation of Wnt target genes and controls cancer stemness. These pathways are related to cell survival, apoptosis, and invasion. But the fact that the WNT pathway controls the stemness suggests that miRNAs related to the WNT pathway may be examined for treatment response. miRNAs which associated with the WNT pathway would be more important for the treatment response while CCKR and p53 pathway associated miRNAs are more promising for the diagnosis.

## 4. Conclusions

To conclude, urinary proteins and miRNAs are promising biomarkers for non-invasive bladder cancer diagnosis and prognosis. To date, urinary *IL-8*, *MMP-9* and *VEGF*, miR-200 family, miR-146 and miR-155 were found to be strongest putative urinary markers. Increased expressions in BC samples and decrease in urinary concentrations after transurethral resection suggests that urinary miR-146a and 155 are mostly originated from cancer cells. However, there are some deficiencies in the methods, overcoming these will lead to a new era for non-invasive bladder cancer diagnosis. ELISA is a widely used method for the analysis of proteins in the urine, either total protein or creatinine concentration is used for normalization and different normalization strategies can affect the results. RNU-48, miR-16, U-6, or total RNA concentrations could be used for normalization in analysis of urine miRNA concentrations, but stable expression of urinary microRNAs in the healthy individuals has not yet been fully explored. Housekeeping miRNAs in the urine should be analyzed in multicentre, large cohort studies and the diagnostic value of urinary miRNAs should be reassessed, because inappropriate normalization causes unreliable results. Urinary protein and microRNA expressions that are isolated and normalized by standardized methods will be highly valuable in terms of non-invasive diagnosis of bladder cancer.

Another issue that should be standardized in the use of urine biomarkers for bladder cancer is the collection and storage of urine specimens until analysis; different groups collect and store samples under different conditions. Some groups collect the first urination in the morning, some prefer any urination during the day, and some collect after bladder stimulation massage. Some groups centrifuge the urine samples before storage at high speed, while some groups use low speed to remove cell debris. Although some studies state that urine fraction does not affect the outcome, use of single or multiple biomarkers does; the lack of homogeneity in terms of urine fraction is also worth considering [79].

Different urine fractions were used in different studies. miR-126, 182, 520e, 618, 122-5p, 16, 21, 34a, 200c, 205, and 211 were found to be up regulated in whole urine samples [53,80,81,82]. Urine sediments are also widely used for miRNA analysis, miR-96, 183, 15b, 1224-3p, 222, 452, 141, 200a/b/c, 18a, 25, 187, 92a, 210, and 141 are up regulated in urine sediments of bladder cancer patients [63,83,84,85,86]. The urine fraction to be used should be well assessed, along with several advantages relative to each other. Numerous population studies will contribute to the development of urinary biomarkers in which, protein, and miRNA expressions are examined in different urinary fractions where a high number of patients and volunteers are involved. These studies will be the beginning of a new era in non-invasive diagnosis and follow-up of bladder cancer.

## Figures and Tables

Vesicular miRNAs originate from apoptotic cells, exosomes or microvesicles. Free miRNAs either could be found as naked forms or ribonucleoprotein complexes coupled with Argonauteargonaute, *HDL*, and nucleoplasmin.

**Table 1:** Urinary proteins for bladder cancer diagnosis.

Abbreviation	Name	Cellular Function and Pathway	Concentration in BC Urine Samples
*A1AT/(SERPINA1)*	Serpin family A member 1	Complement and coagulation cascades [15].	Both ELISA and Multi-array assay revealed that candidates including *A1AT* could discriminate BC patients from controls, NMIBC from MIBC [16]. Strongly correlated with urinary hemoglobin (Spearman’s correlation coefficient >0.8), so the increase in concentration in BC patients’ urine samples may be due to hematuria [11].
*ANG*	Angiogenin	Angiogenesis [17].	Urinary concentrations of *ANG* were significantly elevated in subjects with BC compared to controls [18]. Both ELISA and Multi-array assay revealed that candidates including *ANG* could discriminate BC patients from controls, NMIBC from MIBC [16].
*Apo-A1*	Apolipoprotein A1	Lipid metabolism and transport [19].	Increased in urine samples of aggressive Bladder transitional cell carcinoma (BTCC) compared to low malignant BTCC [5]. Western blot analysis and ELISA assays also revealed that candidates including *Apo-A1* levels in urine samples of BC patients were significantly elevated [4].
*APOE*	Apolipoprotein E	Transport of cholesterol and other lipids [20]	Urinary concentrations of *APOE* were significantly elevated in subjects with BC compared with controls but diagnostic performance of *APOE* as a single biomarker was not sufficient [11,21]. *APOE* levels were significantly increased in high compared to low grade tumors as well as in MIBC compared to NMIBC, both ELISA and Multi-array assay revealed that *APOE* could discriminate BC patients from controls [16,18].
*BTA/hCFHrp*	complement factor H-related protein	Involved in complement regulation.	Urinary concentrations of *BTA* were significantly elevated in subjects with BC [10,11,22,23,24,25].
*CA9*	carbonic anhydrase 9	Involved in pH regulation, control of cell proliferation and transformation [26]. There are also studies advocating axiom.	Urinary concentrations of *CA9* were significantly elevated in subjects with BC [11,24]. But some studies advocating the opposite [18].
*CCL18*	C-C motif chemokine ligand 18	Involved in immunoregulatory and inflammatory processes [27].	Urinary concentrations were significantly elevated in subjects with BC [22]. The concentrations of *CCL18* in urine samples are correlated with hemoglobin, so the increase in concentration in BC patients’ urine samples may be due to hematuria [11].
*CD44*	*CD44* antigen (Epican)	Cell-surface glycoprotein, involved in cell–cell interactions, cell adhesion and migration [28].	Urinary concentrations of *CD44* were significantly decreased in subjects with BC [11]. *CD44* improved the prediction power of *CCL18*, *PAI-1* (AUC = 0.938) [22].
*IL-8*	Interleukin-8	Chemotactic factor, released from several cell types in response to an inflammatory stimulus [29].	Group of proteins, including *IL-8*, had significantly higher expression in BC subjects, *IL-8* was the most consistent one, multivariate regression analysis revealed that only *IL-8* was an independent factor for the detection of BC [25]. Urine *IL-8 l*evels were significantly increased in high compared to low-grade tumors as well as in MIBC compared to NMIBC. *IL8* could discriminate BC patients from controls, low grade BC from high grade BC, NMIBC from MIBC [18].
*MMP-10*	Stromelysin-2/Matrix metalloproteinase-10	Breakdown of extracellular matrix [30].	Significantly higher expression was detected in urine samples of bladder cancer patients compared to healthy controls [11,18]. Both ELISA and Multi-array assay revealed that *MMP-10* could discriminate BC patients from controls, low grade BC from high grade BC, NMIBC from MIBC [16].
*MMP-9*	Matrix metalloproteinase-9	Involved in breakdown of extracellular matrix and in leukocyte migration [31].	Significantly higher expression was detected by ELISA in urine samples of bladder cancer patients compared to healthy controls [11]. Both ELISA and Multi-array assay revealed that *MMP-9* could discriminate BC patients from controls, NMIBC from MIBC [16]. Biomarker levels were also compared with respect to tumor grade and stage. *IL-8*, *MMP-9*, *PAI-1* and *APOE* were significantly increased in high compared to low grade tumors as well as in MIBC compared to non-MIBC [18].
*OPN (SPP1)*	Osteopontin	Appears to be involved in tumorigenesis, metastasis, Cell-matrix interaction and type 1 immunity [32].	Urinary concentrations of *OPN* were significantly elevated in subjects with BC [11].
*PAI-1 (SERPINE1)*	Plasminogen activator inhibitor-1	Inhibitor of fibrinolysis. High expressions are associated with thrombophilia [33].	Urinary concentrations of *CCL18*, *PAI-1*, and *BTA* were significantly elevated in subjects with BC [22]. Urinary concentrations of 14 biomarkers including *PAI-1* were significantly elevated in subjects with BC [11]. Both ELISA and Multi-array assay revealed that *PAI1* could discriminate BC patients from controls, low grade BC from high grade BC, NMIBC from MIBC, *PAI-1* was the most accurate single biomarker for BC followed closely by urinary *IL8* [16].
*PTX3*	Pentraxin 3	Expressed in numerous tissues, such as monocytes, dendritic cells, takes role in inflammation [34].	Urinary concentrations of *PTX3* were significantly elevated in subjects with BC [11]. *PTX* concentration in urine samples analyzed by ELISA but no significant difference found between BC and controls [23].
*Scd-1*	Syndecan-1	Involved in cytoskeleton regulation and exosome biogenesis [35].	Elevated in the urine samples of BC patients but results were not consistent. Any significant association between cancer, control or stage found [11,16,36].
*sFAS*	Soluble *Fas*	Prevents apoptosis induction, and enhances the immunosuppressive effects of tumors [37].	The urinary s*Fas* levels were significantly higher in the patients with UC than in those without cancer [37], significantly elevated in the NMIBC cases with a higher stage or grade or high-risk group category than in those with a lower stage or grade or low-risk group category [38].
*VEGF*	Vascular endothelial growth factor	Active in angiogenesis, vasculogenesis and endothelial cell growth [39].	Urinary concentrations significantly elevated in subjects with BC [11]. *VEGF* was the most accurate urinary biomarker [24] and could discriminate BC patients from controls, NMIBC from MIBC [16].

BC: Bladder cancer; NMIBC: Non muscle invasive bladder cancer; MIBC: Muscle invasive bladder cancer; UC: Urothelial cancer.

**Table 2:** Deregulated urinary miRNAs in bladder cancer.

let-7b	miR-27b	miR-125b	miR-182	miR-222	miR-515
let-7i	miR-29a	miR-126-5p	miR-183	miR-223	miR-520e
miR-1	miR-29a-3p	miR-133a	miR-187	miR-302d	miR-545
miR-7-5p	miR-34a	miR-133b	miR-191	miR-325	miR-556
miR-9-3	miR-92a	miR-134	miR-192	miR-328	miR-589
miR-10a	miR-93	miR-135b	miR-193a-3p	miR-328	miR-616
miR-10b	miR-96	miR-137	miR-200a	miR-335	miR-618
miR-15a	miR-99a	miR-140-5p	miR-200a-3p	miR-338-3p	miR-873
miR-15b	miR-100	miR-141	miR-200b	miR-375	miR-890
miR-16	miR-101	miR-142-3p	miR-200c	miR-377	miR-892a
miR-18a	miR-106b	miR-143	miR-203	miR-423-5p	miR-923
miR-18a-3p	miR-122-3p	miR-145	miR-204	miR-424	miR-940
miR-21	miR-122-5p	miR-146a-5p	miR-205	miR-429	miR-1207-5p
miR-22-3p	miR-124-2	miR-148a	miR-210	miR-451a	miR-1224
miR-24-1	miR-124-3	miR-149	miR-211	miR-452	miR-1224-3p
miR-25	miR-1255b	miR-152	miR-212	miR-483-5p	miR-1225-5p
miR-26a	miR-1255b-5p	miR-155	miR-214	miR-505	
miR-27a	miR-125a	miR-156	miR-221	miR-509	

**Table 3:** 6 miRNAs that target 17 genes among 32 genes.

Validated Target Gene	miRNA
*BCL2*	hsa-miR-100-5p hsa-miR-122-5p hsa-miR-155-5p hsa-miR-29a-3p hsa-miR-429 hsa-miR-21-5p
*CCND1*	
*CCND2*	
*CDK6*	
*CDKN1A*	
*ERBB2*	
*IGF1R*	
*MYB*	
*MYC*	
*PTEN*	
*RECK*	
*RHOA*	
*SMAD4*	
*SP1*	
*TP53*	
*VEGFA*	
*WEE1*	

BCL2: Bcl2 apoptosis regulator; CCND1: Cyclin D1; CCND2: Cyclin D2; CDK6: Cyclin dependent kinase 6; CDKN1A: Cyclin dependent kinase inhibitor 1A; ERBB2: erb-b2 receptor tyrosine kinase 2; IGF1R: Insulin like growth factor 1 receptor; MYB: MYB proto-oncogene; MYC: MYC proto-oncogene; PTEN: Phosphatase and tensin homolog; RECK: Reversion inducing cysteine rich protein with kazal motifs; RHOA: Ras homolog family member A; SMAD4: SMAD family member 4; SP1: Sp1 transcription factor; TP53: Tumor protein p53; VEGFA: Vascular endothelial growth factor A; WEE1: WEE1 G2 checkpoint kinase.

## Supplementary Materials

The following are available online at http://www.mdpi.com/2076-3271/6/4/113/s1, Table S1: Targets of miRNAs. According to “mirBase Catalogue by Experimental Evidences” there are 1443 target genes with strong evidence; Table S2: Cellular pathways associated with target genes were investigated by the Panther Database.
